# Outcomes of patients with initially locally advanced pancreatic adenocarcinoma who did not benefit from resection: a prospective cohort study

**DOI:** 10.1186/s12885-020-6690-1

**Published:** 2020-03-12

**Authors:** Jonathan Garnier, Jacques Ewald, Ugo Marchese, Marine Gilabert, Simon Launay, Laurence Moureau-Zabotto, Flora Poizat, Marc Giovannini, Jean-Robert Delpero, Olivier Turrini

**Affiliations:** 1 Department of Surgical Oncology, Institut Paoli-Calmettes, Marseille, France; 2 Department of Oncology, Institut Paoli-Calmettes, Marseille, France; 3 Department of Radiotherapy, Institut Paoli-Calmettes, Marseille, France; 4 Department of Pathology, Institut Paoli-Calmettes, Marseille, France; 5 Department of Endoscopy, Institut Paoli-Calmettes, Marseille, France; 6 Department of Surgery, Aix-Marseille University, Institut Paoli-Calmettes, CRCM, Marseille, France

**Keywords:** Pancreatic cancer, Locally advanced, Chemotherapy, Chemoradiation, Survival

## Abstract

**Background:**

The current study aimed to evaluate the outcomes of patients with unresectable non-metastatic locally advanced pancreatic adenocarcinoma (LAPA) who did not benefit from resection considering the treatment strategy in the clinical settings.

**Methods:**

Between 2010 and 2017, a total of 234 patients underwent induction chemotherapy for LAPA that could not be treated with surgery. After oncologic restaging, continuous chemotherapy or chemoradiation (CRT) was decided for patients without metastatic disease. The Kaplan–Meier method was used to determine overall survival (OS), and the Wilcoxon test to compare survival curves. Multivariate analysis was performed using the stepwise logistic regression method.

**Results:**

FOLFIRINOX was the most common induction regimen (168 patients, 72%), with a median of 6 chemotherapy cycles and resulted in higher OS, compared to gemcitabine (19 vs. 16 months, hazard ratio (HR) = 1.2, 95% confidence interval: 0.86–1.6, *P* = .03). However, no difference was observed after adjusting for age (≤75 years) and performance status score (0–1). At restaging, 187 patients (80%) had non-metastatic disease: CRT was administered to 126 patients (67%) while chemotherapy was continued in 61 (33%). Patients who received CRT had characteristics comparable to those who continued with chemotherapy, with similar OS. They also had longer progression-free survival (median 13.3 vs. 9.6 months, HR = 1.38, 95% confidence interval: 1–1.9, *P* < .01) and limited short-term treatment-related toxicity.

**Conclusions:**

The median survival of patients who could not undergo surgery was 19 months. Hence, CRT should not be eliminated as a treatment option and may be useful as a part of optimised sequential chemotherapy for both local and metastatic disease.

## Background

Patients diagnosed with non-metastatic locally advanced pancreatic adenocarcinoma (LAPA; comprising both borderline and unresectable tumours) at the time of initial staging receive induction chemotherapy. During the last decade, pancreatic surgeons have been able to perform complex resections/reconstructions in patients with LAPA owing to the benefits of the FOLFIRINOX regimen, which seems to increase the number of patients who finally underwent resection [1]. We recently showed that patients with LAPA strongly benefit from resection after induction chemotherapy^,^ even if complex venous resection/reconstruction is needed [2, 3]. However, most patients who receive induction chemotherapy will not have the opportunity to undergo resection because of progressive disease at restaging, altered performance status, or contraindications during explorative laparotomy (i.e. affirmation of extra-pancreatic disease or major vasculature invasion). In these patients, chemotherapy and chemoradiation (CRT) are the two commonly used treatment modalities administered by oncologists, in combination with new drugs in the setting of prospective trials. To date, no consensual strategies have been defined among patients who cannot benefit from surgery. Patients with extra-pancreatic disease dissemination at restaging or after laparotomy continue with chemotherapy. However, there is no strong evidence about which treatment is optimal for patients with non-metastatic LAPA. Consequently, multidisciplinary physicians have to choose between continuing with chemotherapy (if well tolerated) or CRT according to induction chemotherapy tolerance, patient status, and centre/oncologists preferences.

This study aimed to evaluate the outcomes, according to treatment, among patients with LAPA who did not benefit from resection after induction chemotherapy in clinical settings to provide a picture of the current landscape.

## Methods

### Patient selection

Between January 1, 2010 and December 31, 2017, 342 patients were diagnosed with LAPA (according to the National Comprehensive Cancer Network guidelines [4]), and received induction chemotherapy at the Institut Paoli-Calmettes, Marseille, France. Among these patients, 234 could not benefit from resection and were included in the study; 108 patients underwent pancreatectomy and their outcomes will be reported separately. Patient data were entered prospectively into a clinical database, as approved by the institutional review board and analysed retrospectively. The study participants provided informed consent, and the study protocol adhered to the tenets of the Declaration of Helsinki. The data were gathered according to the CHIRPAN (CNIL n°Sy50955016U) institutional database labelled by InCa (National Institute for Cancer). Approval from all participants was verbal for this study but written for the clinical database CHIRPAN.

### Initial staging and induction chemotherapy

All patients had histologically proven pancreatic adenocarcinoma before any treatment. The initial staging consisted of a physical examination, thoraco-abdominal computed tomography (CT), and CA 19–9 serum level determination. Until 2015, patients did not undergo routine liver magnetic resonance imaging (MRI); positron emission tomography (PET) was not performed routinely owing to the lack of evidence of its relevance in this setting. The chemotherapy regimen (i.e. FOLFIRINOX or gemcitabine) was selected according to the patients’ age, performance status (PS) score, and decision of the multidisciplinary staff. The FOLFIRINOX regimen comprised a combination of oxaliplatin (85 mg/m^2^), irinotecan (180 mg/m^2^), leucovorin, and 5-fluorouracil (bolus and continuous infusion); the cycle was repeated every 2 weeks. If required, the administration of a 5-fluorouracil bolus was stopped and oxaliplatin/irinotecan doses were reduced. The gemcitabine regimen comprised a dose of 1000 mg/m^2^ that was delivered weekly for 3 weeks over subsequent 4-week courses. One cycle corresponded to a 4-week period of treatment. Primary or secondary prophylaxis of neutropenia was initiated using the granulocyte colony-stimulating factor at the physician’s discretion.

### Treatment after restaging

All patients were restaged after 3 months of induction chemotherapy to identify patients with severe adverse events and/or disease progression, which helped determine whether patients should continue with the same regimen. After restaging, three strategies were debated among explorative laparotomy, continuous chemotherapy with or without drug switching, or CRT. CRT comprised intensity-modulated fractionated radiotherapy combined with concurrent chemotherapy, with capecitabine (800 mg/m^2^ twice daily, 5 days/week); the total dose of radiotherapy was 54 Gy in 30 fractions/6 weeks. The decision between CRT and continuous chemotherapy with or without drug switching in patients without evidence of extra-pancreatic disease was associated with the time at which the patients were included. Between 2010 and 2015, our strategy was to perform CRT after induction treatment for local treatment in patients without extra-pancreatic extension and as part of reserve chemotherapy in case of metastatic progression. However, since 2016, according to the results of the LAP07 randomised trial [5], continuing with chemotherapy was the preferred strategy, with at least 8 cycles of chemotherapy being delivered along with a regimen change if the induction treatment was not well tolerated. The surgical decision for explorative laparotomy was made by the multidisciplinary staff. Resection was not performed in the following cases: a) metastatic spread discovered during explorative laparotomy (i.e., liver metastasis, carcinomatosis, and para-aortic lymph node invasion proven on frozen section [6, 7]); b) superior mesenteric artery resection was needed; and c) venous reconstruction was not technically feasible.

### Study parameters

The following variables were evaluated: age, sex, body mass index, serum CA 19–9 level (at diagnosis and after jaundice resolution), PS score (0, 1, 2, or 3), weight loss > 5% body weight, recent diabetes diagnosis (< 1 year), tumour location (i.e. head, body, and tail), back pain, jaundice, arterial invasion at diagnosis, borderline or locally advanced tumours, induction regimen, and continuing with treatment after restaging. The Response Evaluation Criteria in Solid Tumours were not used in our series. The toxicities were evaluated at the start of each cycle, considering the patient history, examination results, PS score, complete blood count, and serum marker results. We documented only the clinically relevant adverse effects, i.e., the grade 3 and 4 toxicities considering the four most frequents adverse events—leucopenia, diarrhoea, polyneuropathy, and infectious complications—by reviewing the patient charts. The adverse events were graded according to the National Cancer Institute Common Terminology Criteria for Adverse Events [8]. The *BRCA* mutation status was not routinely evaluated during the period of inclusion.

### Statistical analysis

Data analyses were performed using GraphPad Prism version 6 (GraphPad Software Inc., La Jolla, CA, USA) and SPSS® version 24 (SPSS Inc., Chicago, IL, USA). Categorical factors were compared using Fisher’s exact test or the chi-squared test, as appropriate. Continuous variables were assessed using the student’s *t*-test. The associations between the categorical factors and overall survival (OS) were assessed using the Kaplan–Meier method (based on the date of diagnosis and censoring date, January 1, 2019). Among patients with rapid disease progression and early risk of death, we compared the survival curves using the Wilcoxon test, and obtained the hazard ratio (HR) and 95% confidence interval (CI). Multivariate analysis was performed using stepwise logistic regression. Statistical significance was set at *P* < .05.

## Results

### Induction treatment, restaging, and strategy after restaging

The patient characteristics are summarised in Table 1. FOLFIRINOX was the most commonly used induction regimen (168 patients, 72%), with a median number of 6 cycles of chemotherapy. Patients who received FOLFIRINOX were younger (*P* < .01) and had a better PS score (*P* < .01) compared to patients who received gemcitabine (Table 2). At restaging, 187 patients (80%) had non-metastatic LAPA whereas 47 (20%) showed metastatic disease progression. Among patients with non-metastatic LAPA, 126 patients (67%) received CRT whereas 61 (33%) continued with chemotherapy (Table 3). Metastatic disease progression was significantly more frequent among patients who received CRT (90 patients, 71%) than among those who continued with chemotherapy (26 patients, 43%; *P* < .01), with a comparable mean delay (12 vs. 10 months; *P* = ns).

**Table 1:** Characteristics of patients with LAPA treated with induction chemotherapy (*n* = 234)

**Sex ratio (M/F)**	2 (121/103)	
**Mean age (range)**	67 (29–89)	
**Performance status**^**a**^**(%)**		
0–1	215 (92)	
2–3	19 (8)	
**Mean BMI**^**a**^**(range)**	24 (14–42)	
**Weight loss > 5% body weight**^**a**^**(%)**	160 (68)	
**Diabetes (%)**	15 (36)	
**Back pain**^**a**^**(%)**	152 (65)	
**Jaundice (%)**	136 (84)^**b**^	
**CA 19–9 serum level**^**a**^**(UI) (range)**	2292 (7–88,300)	
**Tumour location (%)**		
Head	161 (69)	
Body	57 (24)	
Tail	16 (7)	
**Tumour classification (%)**		
Borderline	158 (68)	
Locally advanced	76 (32)	
**Arterial invasion**^**a**^**(%)**	93 (40)	
**Induction regimen (%)**		
FOLFIRINOX	168 (72)	
Gemcitabine	66 (28)	
**Mean number of cycles before restaging (range)**	6 (4–8)	
**Explorative laparotomy (%)**	45 (19)	
**Treatment after restaging/laparotomy (%) and mean number of cycles (range) after restaging/laparotomy**		
FOLFIRINOX	78 (33)	4 (0–18)
Gemcitabine	30 (12)	4 (0–15)
Chemoradiation	126 (54)	2 (0–19)

*LAPA* locally advanced pancreatic adenocarcinoma, *BMI* body mass index

^a^at diagnosis

^b^calculated for patients with head LAPA

**Table 2:** Characteristics of patients with LAPA at initial staging and according to the delivered induction regimen (*n* = 234)

	FOLFIRINOX (***n*** = 168)	Gemcitabine (***n*** = 66)	***P-***value
**Mean age** **+** **SD**	64 ± 9.55	75 ± 8.76	***< 0.01***
**Performance status score 2–3 (%)**	8 (5)	11 (17)	***< 0.01***
**Mean BMI** **+** **SD**	23.5 ± 3.81	23.5 ± 4.41	ns
Weight loss > 5% body weight * (%)	110 (65)	50 (76)	ns
**Diabetes (%)**	10 (6)	5 (7.5)	ns
**Back pain (%)**	108 (64)	44 (67)	ns
Serum CA 19–9 level* (IU) (+SD)	3884 ± 13,137	1743 ± 4916	ns
**Tumour location (%)**			ns
Head	117 (70)	44 (65)	
Body	40 (24)	17 (26)	
Tail	11 (6)	5 (9)	
**Tumour classification (%)**			ns
Borderline	116 (69)	42 (64)	
Locally advanced	52 (31)	24 (36)	
**Arterial invasion (%)**	68 (40.5)	25 (38)	ns

*LAPA* locally advanced pancreatic adenocarcinoma, *BMI* body mass index

**Table 3:** Characteristics of patients with non-metastatic LAPA at restaging, according to the delivered treatment, CRT, or continued chemotherapy (*n* = 187)

	CRT (***n*** = 126)	Chemotherapy (***n*** = 61)	***P-***value
**Mean age** **+** **SD**	66.1 ± 9.85	71.4 + 9.97	***< 0.01***
**Performance status**^**a**^**(%) ≥ 2**	7 (5.6)	8 (13)	**0.089**
**Mean BMI** **+** **SD**	23.1 ± 3.77	23.2 ± 4.78	**0.89**
**Weight loss > 5% body weight**^**a**^**(%)**	92 (73)	42 (69)	**0.55**
**Diabetes (%)**	19 (15)	8 (13)	**0.72**
**Back pain**^**a**^**(%)**	88 (70%)	38 (62%)	**0.3**
**Serum CA 19–9 level**^**a**^**(IU) (range)**	2180 ± 5718	1886 ± 4785	**0.74**
**Tumour location (%)**			
Head	82 (65)	43 (70)	**0.46**
Body	36 (29)	16 (26)	**0.66**
Tail	8 (6.3)	2 (3.3)	**0.5**
**Arterial invasion**^**a**^**(%)**	63 (50)	21 (34)	***0.045***
**Continued regimen (%)**			
FOLFIRINOX		29	
Gemcitabine		32	
5 FU	126		
**Mean number of cycles after restaging (range)**			
**Toxicity**			
Grade ≥ 3	7 (5.5)	15 (25)	***< 0.01***
Neutropenia	10 (8)	20 (33)	***< 0.01***
Diarrhoea	6 (5)	4 (7)	***Ns***
Polyneuropathy	0	10 (16)	***< 0.01***

*LAPA* locally advanced pancreatic adenocarcinoma, *BMI* body mass index

^a^at diagnosis

### Survival

**Fig. 1:**
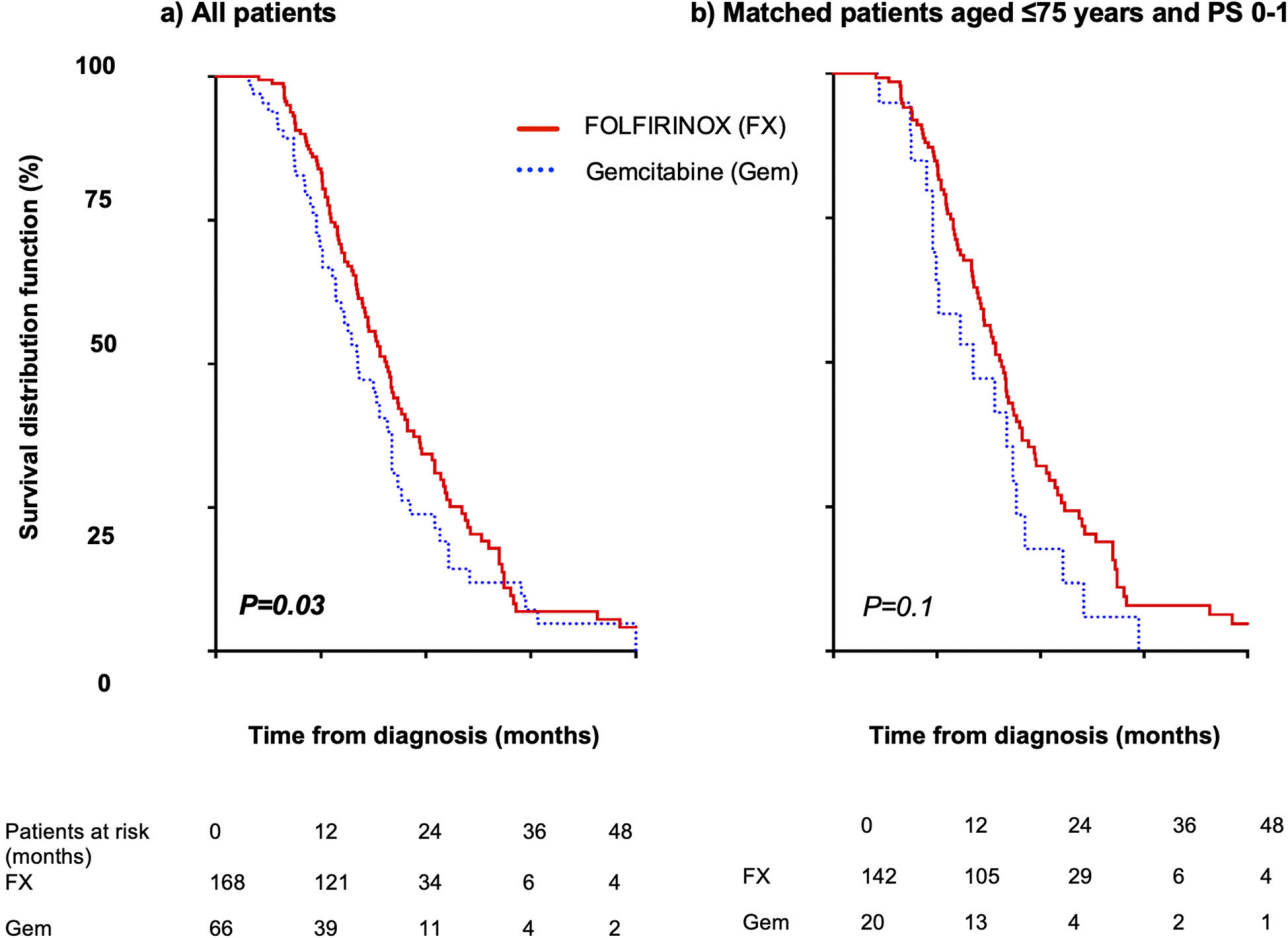
Overall survival of patients with LAPA who received FOLFIRINOX or gemcitabine as induction chemotherapy: **a** overall survival of all patients, and **b** overall survival of patients aged ≤75 years and performance status (PS) score of 0–1. LAPA, locally advanced pancreatic adenocarcinoma

**Fig. 2:**
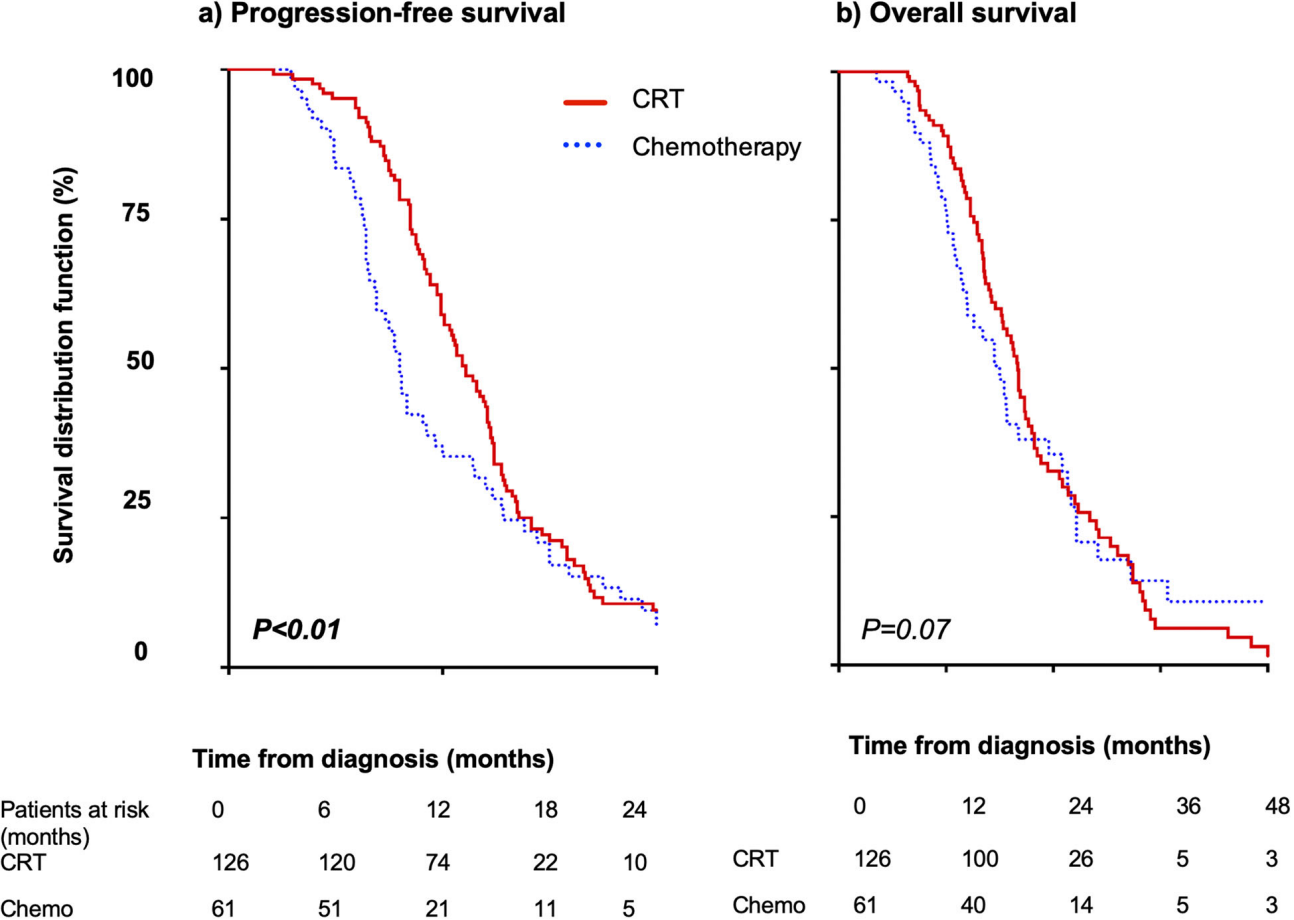
Survival of patients with non-metastatic LAPA at restaging (*n* = 187) who received CRT or continued chemotherapy. LAPA, locally advanced pancreatic adenocarcinoma; CRT, chemoradiation

No patient was lost to follow-up (mean follow-up duration, 31 months). During the study period, 70% of patients (*n* = 163) had metastatic progression, with the liver being the more frequent site of metastasis (*n* = 88, 54%) followed by carcinomatosis (*n* = 45, 28%). The median survival time of all patients was 18.7 months; the 1-, 3-, and 5-year OS rates were 80, 7, and 1%, respectively. At diagnosis, degraded PS, weight loss, and CA 19–9 level > 500 U/mL were independent factors that poorly influenced OS (Table 4). A minimum of 8 cycles of chemotherapy were needed to obtain the optimal OS (19.6 vs. 18.4 months) without statistical significance (*P* = .36). On comparing induction chemotherapy regimens, FOLFIRINOX resulted in a higher OS than gemcitabine did (19 vs. 16 months, HR = 1.2, 95% CI: 0.86–1.6, *P* = .03) (Fig. 1a). However, no difference was observed after adjusting for age (≤75 years) and PS score (0–1) (Fig. 1b). At restaging, patients with non-metastatic disease (*n* = 187) who received CRT or continued with chemotherapy had similar patient characteristics (except for age, wherein patients in the chemotherapy group were aged > 75 years; 18% vs 43%, *P* < .01) and tumour characteristics (except for arterial invasion) as well as CA 19–9 levels. Although patients who received CRT showed longer progression-free survival (median 13.3 vs. 9.6 months, HR = 1.38, 95% CI: 1–1.9, *P* < .01) (Fig. 2a), the benefit of CRT on progression-free survival disappeared (*P* = .17) when adjusted for age < 75 years. Patients with CRT had metastatic disease more frequently (HR = 2.9, 95% CI: 1.5–5.4, *P* < .01) and a trend of prolonged OS (median 20 vs. 18 months, *P = .07*) (Fig. 2b) compared to those who continued with chemotherapy.

**Table 4:** Univariate and multivariate analyses of factors influencing overall survival at diagnosis (*n* = 234)

	***P-***value	Hazard ratio [95% CI]	***P-***value
**Performance status score ≥ 2**	< 0.01	1.92 [1.06–3.46]	***0.031***
**Borderline status at diagnosis**	0.35		ns
**Arterial invasion at diagnosis**	0.48		ns
**Back pain**	0.041		ns
**Weight loss**	0.037	1.49 [1.02–2.19]	***0.041***
**CA 19–9 level > 500 IU**	0.087	1.79 [1.25–2.57]	***< 0.01***
**Continuous chemotherapy**	0.065		ns

## Discussion

The current study showed that the clinical status at diagnosis strongly influenced OS rather than the chemotherapy regimen, and that patients without metastatic disease at restaging seemed to benefit from CRT.

### Patient characteristics and clinical status

LAPA often influences the clinical status of patients with weight loss and poor PS score [9], indicating that nutrition and supportive care might help patients receive an optimal therapeutic strategy (i.e. induction treatment and resection). In the present study, we confirmed the importance of clinical status (PS score and weight loss), as it was an independent factor of poor OS, along with the serum CA 19–9 level. Thus, a high initial CA 19–9 level might indicate metastatic disease, with a reduced OS of 12 months [10].

### Induction treatment

CRT as induction treatment for patients with LAPA was changed to chemotherapy, because CRT could not be consistently used as a local treatment in a large proportion of patients with unknown metastatic disease [11, 12]. Thus, gemcitabine was the preferred induction treatment for patients with LAPA until 2010 [13], which was the landmark treatment in the FOLFIRINOX era [14]. As FOLFIRINOX resulted in more patients being able to undergo resection [1], it also provided a survival advantage over the former gemcitabine regimen in the current study. However, patients who received gemcitabine induction chemotherapy were older or had a weaker clinical status compared to patients who received the FOLFIRINOX regimen. Interestingly, after adjusting for these factors, there was no survival advantage among patients who received the FOLFIRINOX regimen, possibly indicating that the clinical status was more important than the treatment regimen.

### Continuing with chemotherapy or CRT at restaging

Data on the therapeutic strategies and their impact on survival are scarce in patients with unresectable LAPA. In the current study, patients who received CRT at restaging more often developed metastasis, reinforcing the concept that pancreatic cancer is a micrometastatic disease [5, 12]. Our findings are consistent with those of the LAP07 randomised trial that failed to demonstrate any benefit of CRT in patients with LAPA [5].

Nevertheless, CRT had a benefit effect considering the following reasons. First, pancreatic adenocarcinoma shows the expression of different genes [15] and some patients probably had a particular tumour biology that confers radiosensitivity. Accordingly, we performed early neoadjuvant CRT for patients with resectable pancreatic cancer, but abandoned this strategy owing to disappointing results on considering all treated patients [16]. However, we observed that some resected specimens had a complete histologic response, indicating radiosensitivity [17, 18]. Second, in the current study, patients who received CRT had a prolonged progression-free survival and a trend of prolonged OS. This indicates that CRT provided true local control [19], consistent with the loco-regional progression rate of 30% reported previously [15, 20]. This does not result in a significant improvement in OS, as the micrometastatic part of the disease was probably insufficiently treated. Indeed, patients often receive only 4 to 6 cycles of induction chemotherapy before CRT, which therefore needs to be optimised. Third, continuing with chemotherapy results in a rapid increase in the incidence of adverse events, requiring dose reduction or chemotherapy interruption. In the current study, continued chemotherapy was associated with a higher incidence of adverse events, comparable to the results of a previous series [21] probably because among patients who received CRT, treatment was discontinued until relapse was observed. Fourth, CRT should improve with the development of new techniques such as stereotactic body radiation therapy [22, 23] that may provide benefit over conventionally fractionated radiation therapy in the neoadjuvant setting (awaiting the results of the Alliance A021501 trial [24]), and was hence included in the last NCCC version for 2020 considering the treatment for locally advanced pancreatic cancer [25].

### Perspectives in patients with non-metastatic LAPA at restaging

Physicians must focus on restaging to determine the appropriate treatment for each patient, including CT, liver MRI [26], explorative laparoscopy (with para-aortic lymph node picking if technically feasible), and serum CA 19–9 level measurements. After restaging, patients with no evidence of metastatic disease should have a discussion with multidisciplinary staff considering the following four aspects. First, physicians should consider the tumour evolution after induction treatment, as it makes sense to continue with chemotherapy in patients with an objective response. In this setting, ^18^F-flurorodeoxyglucose PET [27–29] imaging was helpful, but the utility has to be validated in large prospective cohorts. Second, the tolerance to induction chemotherapy should be kept in mind, considering the presence of germline mutations [30, 31]. Third, the number of cycles administered: our results showed that patients might benefit from at least 8 cycles of chemotherapy before CRT, similar to the results of other studies [20]. Finally, the level of CA 19–9 should be considered before treatment is switched to CRT, as the current study showed that a CA 19–9 level > 500 U/L was an independent predictive factor of survival, indicating persistent tumour activity. Thus, alternating between chemotherapy and CRT might help in treating both local and metastatic disease.

Oncologists should also use biomarkers, such as changes in the CA 19–9 level [20], neutrophil-to-lymphocyte ratio [32], and liquid biopsies [33–35] to propose tailored treatment after restaging among patients with no evidence of metastatic disease.

### Study limitations

The present study has some limitations. Owing to the retrospective nature of the study, radiologic assessment of the objective response to induction treatment was not precisely noted. In addition, at the time of restaging, non-metastatic disease was affirmed by using mainly CT and eventually explorative laparotomy. Indeed, any patients diagnosed with non-metastatic disease who received CRT probably had an unknown metastatic disease that negatively influenced the OS. Moreover, we did not take into account objective measures of the quality of life. Considering the statistical analyses, we used the Wilcoxon test to compare the survival curves of patients with rapid disease progression and early risk of death. However, when the classic log-rank test was used, no differences were observed. Nevertheless, the recent period of patient inclusion, the large sample size, and the homogeneity of the studied population are strengths that compensate for these limitations.

## Conclusion

The present study evaluated the outcomes of patients with LAPA in a high-volume centre for pancreatic cancer in the clinical setting. CRT resulted in better progression-free survival and a trend of improved OS over continued chemotherapy for patients without any metastasis at restaging and who did not benefit from surgery. Thus, CRT should not be completely discarded as a treatment option, as it might be useful when integrated as part of optimised sequential treatment for both local and metastatic disease. A low CA 19–9 level, a minimum of 8 cycles of chemotherapy, and controlled disease are factors that can indicate the switch to CRT. Moreover, outcomes might improve owing to the use of new-generation radiation therapy.

## Data Availability

Patient data were prospectively entered into a clinical database, as approved by the institutional review board and analysed retrospectively (CHIRPAN). The datasets analysed during the current study are not publicly available but are available from the corresponding author on reasonable request.

## Abbreviations

BMI: Body mass index
CRT: Chemoradiation
CT: Computed tomography
LAPA: Locally advanced pancreatic adenocarcinoma
MRI: Magnetic resonance imaging
OS: Overall survival
PET: Positron emission tomography
PS: Performance status
